# Editorial: Exploring reliable markers and prediction indexes for the progression from subjective cognitive decline to cognitive impairment, volume II

**DOI:** 10.3389/fnagi.2023.1208478

**Published:** 2023-05-11

**Authors:** Frank Jessen, Ying Han, Jiehui Jiang

**Affiliations:** ^1^Department for Neurology and Psychiatry, University of Cologne, Cologne, Germany; ^2^Xuanwu Hospital, Capital Medical University, Beijing, China; ^3^School of Life Science, Shanghai University, Shanghai, China

**Keywords:** preclinical Alzheimer's disease, subjective cognitive decline, marker, neuroimaging, prediction indexes

The early detection of Alzheimer's disease (AD) has become increasingly important since it allows for earlier intervention and perhaps more effective therapy. Subjective cognitive decline (SCD) has been proposed as a crucial stage in the progression of AD (Livingston, 2020). Furthermore, studies have shown that individuals with SCD have a higher risk of progressing to mild cognitive impairment (MCI) and AD. As such, accurately identifying and assessing SCD in individuals may help identify those at risk for developing AD, and allow for early intervention and management. With the development of neuroimaging and molecular techniques, we are now able to explore the early biomarkers, which may lead to the early therapeutic interventions in the future (Yu et al., 2020; Lu et al., 2021). Therefore, the paucity of biomarkers for SCD makes it possible for physicians to predict individual cognitive decline and progression to dementia.

Taking this into consideration, the present Research Topic of “Exploring Reliable Markers and Prediction Indexes for the Progression from Subjective Cognitive Decline to Cognitive Impairment–Volume II” by Frontiers in Aging Neuroscience provides valuable insights and fresh perspective on this critical issue, which has been covered in seven papers. These papers offer updates on the risk factors and/or protective factors associated with the progression from SCD to cognitive impairment using various methods such as epidemiological methods, neuroimaging techniques and so on.

Liu et al. investigated the potential link between SCD and the development of postoperative delirium (POD). In addition, they explored the possible involvement of amyloid beta (Aβ) and tau protein in cerebrospinal fluid (CSF). The findings obtained from multivariate logistic regression analysis indicated that SCD and phosphorylated tau (P-tau) were identified as significant risk factors for POD. Furthermore, the study revealed that the association between SCD and POD was partially mediated by P-tau.

Impaired metamemory capacity, which refers to the ability to monitor and regulate one's memory processes, may portend a preclinical stage of progressive AD. To investigate the changes of metamemory along the AD continuum, Li Q. et al. recruited individuals who were cognitively unimpaired, those with SCD, MCI and AD patients. Based on degree of confidence (DOC) of discrepancies between judgments and memory performance, they reported higher DOC in the SCD Aβ+ group. In addition, there was an increasing trend of overconfidence with the decline of cognition along the AD continuum.

Tzeng et al. conducted a study to investigate whether the clinical dementia rating (CDR) scale could be used to predict disease progression to dementia or reversion to normal cognition. They focused on the sum of boxes of the CDR and used a Cox regression model to analyze their results. The study found that conversion rates increased as CDR-SB scores increased. The researchers also found that patients with CDR-SB scores of zero had a lower risk of dementia conversion and a higher likelihood of reversion to normal cognition.

Serum klotho conducted a study using cross-section data from 2,171 participants to investigate the potential relationship between serum klotho, an anti-aging protein, and cognitive performance. The results suggest that serum klotho may serve as a marker for cognitive health and thus warrants further attention (Linghui et al.).

Research from Li K. et al. reported that patients with elevated levels of cystatin C were at a greater risk of developing MCI. In addition, they found that glucose homeostasis indicators play a mediating role in the relationship between serum cystatin C and MCI.

Based on path analysis, Kuroda et al. explored the relationship across cerebral white matter lesions (WML), regional cerebral blood flow (rCBF) and cognitive decline. The results revealed interrelationships among the lateral ventricular, periventricular WML (PvWML-V), and rCBF of the anterior cingulate gyrus. Furthermore, a direct relationship was observed between PvWML-V and cognitive decline.

Subcortical ischemic vascular disease (SIVD) is a significant contributor to vascular cognitive impairment. Tan et al. focused on the association between changes in gray matter volume and cognitive impairment in patients with SIVD, providing evidences for the early diagnosis of cognitive impairment symptoms caused by cerebrovascular disease.

Overall, this topic presents several new findings and insights on potential biomarkers for SCD, which may aid physicians better to predict individuals' cognitive decline and progression to dementia. These biomarkers have the potential to revolutionize the diagnosis and treatment of SCD, enabling earlier intervention and improving patient outcomes.

## Author contributions

FJ, YH, and JJ have written this editorial for the Research Topic they have edited. All authors contributed to the article and approved the submitted version.
